# Effectiveness of Fit and Trimmed Staffs (FATS) program on weight management among the healthcare providers at Simpang Health Clinic, Perak: A pre-post interventional study

**DOI:** 10.51866/oa.107

**Published:** 2022-10-18

**Authors:** Shing Ni Leow, Chai Li Tay, Wei Wei Ng, Mior Nurshafiq Mior Mohammad Jafri

**Affiliations:** 1 MD (MMA, Russia) , Doctor of Family Medicine (UKM), Klinik Kesihatan Changkat Jering, Changkat Jering, Taiping, Perak, Malaysia. Email: snleow@hotmail.com; 2 MD (UKM), MFamMed (UM), Klinik Kesihatan Simpang, Jalan, Kuala Kangsar, Simpang, Taiping, Perak, Malaysia.; 3 BSc (Hon) Nutrition (UKM), Klinik Kesihatan Simpang, Jalan, Kuala Kangsar, Simpang 34700, Taiping, Perak, Malaysia.; 4 BSc OT (UiTM), Klinik Kesihatan Simpang, Jalan, Kuala Kangsar, Simpang, Taiping, Perak, Malaysia.

**Keywords:** Weight Reduction Programs, Health personnel, Obesity

## Abstract

**Introduction:**

Obesity is associated with an increased risk for non-communicable diseases. Local studies have shown that 33.1% of healthcare providers (HCPs) are overweight, while 21.1% are obese. Interventions that consist of diet, physical exercise and cognitive behavioural training have been shown to be successful in reducing weight.

**Method:**

We designed a weight loss programme for our HCPs named the ‘Fit and Trimmed Staff programme, which consisted of 3 months of group education on obesity-related health problems led by a doctor, a pharmacist, a nutritionist and an occupational therapist among HCPs. Monthly individual dietary counselling by a nutritionist was also provided for 6 months. We measured the body weight, body mass index, percentage of body fat, visceral fat and percentage of skeletal muscle of the HCPs before and after the intervention.

**Results:**

Forty-five (56.25%) HCPs at Simpang Health Clinic were either overweight or obese; the majority of them were drivers and administrative clerks (100%), followed by health attendants (69.2%) and medical assistants (63.6%). At 6 months post-intervention, there was a trend towards a non-significant reduction in the fat percentage (median=-0.8%, P=0.423). Approximately 42% (n=19) of the HCPs lost weight, while 58% gained weight. Weight loss was observed more commonly in the male HCPs (>50%) than in the female HCPs.

**Conclusion:**

A weight loss programme solely consisting of health discussion and nutritional advice is inadequate to induce weight reductions. A multimodal approach may be considered in managing weight among HCPs.

## Introduction

Obesity is one of the risk factors for developing hypertension, dyslipidaemia, type 2 diabetes mellitus, cardiovascular disease, stroke and cancer. It is also associated with reduced work productivity, physical fitness and muscular strength and increased risk for musculoskeletal pain.^1-3^ Healthcare providers (HCPs) should act as role models, promote public awareness of obesity prevention and advocate a healthy lifestyle among patients. They are commonly thought as being well informed about the risks of obesity. Ironically, studies from Scotland, Botswana and India have consistently found that HCPs have a higher risk of obesity than the general population.^4-6^ A Malaysian study revealed that 33.1% of HCPs were overweight, while 21.1% were obese. Being a nurse was significantly associated with a higher risk of obesity.^7^ Female HCPs also have a higher prevalence of metabolic syndrome than their male counterparts.^4^

A meta-analysis showed that when HCPs provided weight loss advice, it positively influenced patients’ self-efficacy and motivation in weight loss behaviour.^7^ Obesity among HCPs is a barrier in obesity care. A study indicated that HCPs with obesity were less confident in providing healthy nutritional/exercise advice to their patients.^8^ HCPs with a normal body mass index (BMI) perceived that weight loss advice from doctors with overweight/obesity lack trustworthiness and authenticity.^8^ Lack of training/education on weight management among HCPs has led to their inability to accurately estimate calorie and salt contents in food and their unawareness about the amount of exercise required to burn off excess calories.^9^ There is a need to support and educate HCPs to better manage the challenges associated with obesity. This is an imperative that enables HCPs with a normal BMI to advocate for weight management more effectively.

There are multimodal strategies in managing weight among HCPs. Behavioural (e.g. counselling by a trained professional, exercise training session or cooking demonstration) and educational strategies (e.g. lecture or educational material) are able to modify diet and physical activity behaviours of HCPs.^10^ Organisational support strategies, such as provision of incentives, are also effective for weight loss by improving diet and physical activity behaviours.^10^ Environmental strategies, including access to healthier meals, to manage the weight of HCPs via healthy eating behaviours also yield positive results.^11^ Interventions targeting both diet and physical activity behaviours have been successful in improving weight outcomes.^12^ The FINALE-health study indicated that interventions consisting of diet, physical exercise and cognitive behavioural training during working hours for 1 hour/week significantly reduced body weight, BMI and body fat percentage among female HCPs with overweight over 12 months.^13^

Weight management can be challenging among HCPs owing to their busy schedule and lack of motivation. Interventions at the workplace may enhance motivation for weight management. Therefore, we developed the ‘Fit and Trimmed Staff’ (FATS) programme to utilise our workplace as an arena for weight loss initiatives among HCPs with overweight/obesity. The FATS programme is a self-initiated programme by the authors. It consists of 3 months of group education on obesity-related health problems led by a doctor, a pharmacist, a nutritionist and an occupational therapist. Monthly individual dietary counselling by a nutritionist was also provided for 6 months. Owing to manpower constraints during the COVID-19 pandemic, interventions occurred at a 3-4-week interval, rather than at a 1–2-week interval as in other studies. This study aimed to assess the effectiveness of the FATS programme in weight management among HCPs with overweight/ obesity at Simpang Health Clinic and determine the changes in the BMI, percentage of body fat, visceral fat and percentage of skeletal muscle after the FATS programme.

## Methods

This pre—post interventional study consisted of two overlapping phases. In the first phase, we provided HCPs with overweight/ obesity working at Simpang Health Clinic with 3 months of weekly group education on obesity-related health problems. Each session lasted 1 hour and was conducted by a doctor, a pharmacist, a nutritionist and an occupational therapist. The topics covered were obesity and its complications, pharmacotherapy for obesity and its side effects, healthy diet for weight reduction and healthy lifestyle. This phase spanned 3 months (from 1 September to 31 December 2020). In the second phase, monthly individual dietary counselling by a nutritionist was provided, with each session lasting 10–15 minutes. This phase spanned 6 months (from 1 September 2020 to 15 March 2021). The body weight, BMI, percentage of body fat, visceral fat and percentage of skeletal muscle of the HCPs were measured between 1 and 15 September 2020 before the interventions started. The second set of measurements was taken immediately after the intervention between 1 and 15 March 2021.

HCPs who were overweight or obese, working at Simpang Health Clinic between 1 September 2020 and 15 March 2021 and had attended both phases of the intervention were included. HCPs who were on maternity leave, pregnant or on long medical/study leave were excluded. Epi Info version 3.5.4 ( Centers for Disease Control and Prevention (CDC), Atlanta, Georgia (US)). It was used to calculate the sample size based on the following: a) The overweight/obesity rates among public sector HCPs was 54.2% based on a Malaysian study;^14^ b) the total number of HCPs at Simpang Health Clinic during the study period was 80; and c) the confidence level was set at 95% and the significance level at p<0.05. The estimated sample size was 67. The universal sampling method was used. In our setting, 45 (56.25%) HCPs were overweight or obese and thus included in our study.

Our study instruments consisted of an electrical weighing machine (OMRON Body Composition Monitors Model: HBF-214) and a data collection form used to collect information on demographics (age, sex or designation), date of measurement, height, weight, BMI, percentage of body fat, visceral fat and percentage of skeletal muscle.

Recruitment was performed on the basis of the body weight, BMI, percentage of body fat, visceral fat and percentage of skeletal muscle of all 80 HCPs at Simpang Health Clinic. Measurements were conducted by a nutritionist, and results were charted in a pocket-sized data collection form. The data form was kept by the participants themselves; they were encouraged to keep them among their essential belongings, such as in their wallets, to serve as a frequent reminder to watch their weight. All HCPs with overweight/obesity (n=45) identified were invited by the principal investigator to participate in the study. All of them provided written consent. At the end of the study period, data were retrieved from the pocketsized data form kept by the participants.

Data were analysed using the SPSS version 25. Continuous data were plotted onto histograms and tested for normal distribution using the Kolmogorov—Smirnov test. Normally distributed continuous variables, including age, were presented as means with standard deviations. Non-normally distributed data, including weight, BMI, fat percentage, visceral fat and percentage of skeletal muscle, were presented as medians with interquartile ranges. Categorical variables, such as sociodemographic data and BMI, were described as frequencies and percentages. Pre-and post-intervention weight, BMI, percentage of body fat, visceral fat and percentage of skeletal muscle were compared using the Wilcoxon signed rank test. A P-value of <0.05 was considered statistically significant.

## Results

A total of 80 HCPs working at Simpang Health Clinic were screened, among whom 45 (56.25%) were overweight/obese. The majority of the HCPs with overweight/obesity were men (63.6%) and aged between 45 and 56 years (80%). The highest prevalence of overweight/obesity was found among drivers and administrative clerks (100%), followed by health attendants (69.2%) and medical assistants (63.6%). The mean age of the HCPs with overweight/obesity was 39.24±8.33 years

We included the two staff with morbid obesity who were unwilling to consider bariatric surgery despite being counselled about the poor outcomes of diet and education interventions alone. All staff with comorbidities, including diabetes mellitus and hypertension, were followed up under the family medicine specialist in the clinic. No staff had medical/psychiatric conditions that may affect their BMI/weight.

After the intervention, 42% (n=19) of the HCPs lost weight, while 58% (n=26) gained weight. Among those who lost weight, the percentage of change ranged from 0.1% to 9.3%; among those who gained weight, the percentage of change ranged from 0.4% to 15.1%. According to profession, 100% of the pharmacists, 75% of the allied health professionals and 60% of the drivers and administrative clerks successfully lost weight. Weight loss was most commonly observed in those aged 23–34 years (64.7%). Among the 19 HCPs who lost weight, 57.1% (n=8) were men, and 35.5% (n=11) were women. More than 50% and approximately 35.5% had weight loss after the intervention among the male and female HCPs with overweight/ obesity, respectively (**Table 1**).

**Table 1:** Sociodemographic data of the HCPs at Simpang Health clinic (n=80).

Variable	Total no. of HCPs (n=80)	HCPs with overweight/obesity, percentage according to each variable (n=45), n (%)	Weight change after the intervention (n=45)	
			Weight loss (n=19), n (%)	Weight gain (n=26), n (%)
*Designation*				
Doctors	13	7 (53.8)	3 (42.9)	4 (57.1)
Pharmacists	7	3 (42.9)	3 (100.0)	0 (0.0)
Nurses	23	10 (43.5)	3 (30.0)	7 (70.0)
Medical assistants	11	7 (63.6)	2 (28.6)	5 (71.4)
Health attendants	13	9 (69.2)	2 (22.2)	7 (77.8)
Allied health professionals (radiographers, occupational therapists or laboratory technicians	8	4 (50.0)	3 (75.0)	1 (25.0)
Drivers and administrative clerks	5	5 (100.0)	3 (60.0)	2 (40.0)
*Age (year)*				
23-34	35	17 (48.6)	11 (64.7)	6 (35.3)
35-44	30	16 (53.3)	6 (37.5)	10 (62.5)
45-56	15	12 (80.0)	2 (16.7)	10 (83.3)
*Sex*				
Male	22	14 (63.6)	8 (57.1%)	6 (42.9%)
Female	58	31 (53.4)	11 (35.5%)	20 (64.5%)

HCP, healthcare provider

Before the intervention, 53.3% (n=24) of the HCPs with an abnormal BMI were categorised to have overweight; 35.6% (n=16), obesity I; 6.7% (n=3), obesity II; and 4.4% (n=2), obesity III. After the intervention, 2.2% (n=1) of the HCPs who were overweight successfully achieved a normal BMI. There was a twofold increase in the number of participants with obesity II postintervention, contributed by one from the overweight category and two from the obesity I category pre-intervention. Two of the HCPs with obesity III remained in the same classification after the intervention (**Table 2**).

**Table 2:** Classification of BMI pre-intervention and 6 months post-intervention (n=45).

BMI (kg/m^2^)	Classification	Pre-intervention		6 months post-intervention
		Number	Percentage	Number	Percentage
18.5-24.9	Normal	0	0	1	2.2
25.0-29.9	Overweight	24	53.3	22	48.9
30.0-34.9	Obesity I	16	35.6	14	31.1
35.0-39.9	Obesity II	3	6.7	6	13.3
≥40	Obesity III	2	4.4	2	4.4

BMI was classified in accordance with the Malaysian Clinical Practice Guidelines on Management of Obesity (2004) and Malaysian Clinical Practice Guidelines on Management of Type 2 Diabetes Mellitus (6th edition, 2020) adapted from the WHO Consultation Group. BMI, body mass index

There was a 0.8% reduction in the median fat percentage post-intervention; however, the result was not significant (P=0.423). The weight, BMI, fat percentage, visceral fat and muscle percentage did not significantly change post-intervention. The median weight did not significantly change throughout the 6-month study period: pre-intervention, 74.4 kg; first month, 74.4 kg; third month, 75.5 kg; fourth month, 75.4 kg; fifth month, 75.3 kg; postintervention, 74.4 kg. Therefore, only two sets of measurements — pre- and post-programme weight charts — were retrieved in our study (**Table 3**).

**Table 3:** Median weight, BMI, fat percentage, visceral fat and muscle percentage pre- and postintervention (n=45).

Variables	Median (interquartile range)		Z statistic	P-value^*^
	Pre-intervention	Post-intervention		
Weight	74.40 (67.90, 81.95)	74.40 (68.70, 80.85)	-1.321	0.186
BMI	29.20 (26.45, 32.20)	29.20 (26.55, 33.05)	-1.668	0.095
Fat percentage	36.60 (29.85, 39.20)	35.80 (29.85, 39.35)	-0.801	0.423
Visceral fat	12.00 (10.00, 17.25)	13.00 (10.00, 18.00)	-1.861	0.063
Muscle percentage	24.00 (22.35, 29.85)	24.30 (22.45, 30.00)	-0.015	0.988

* Wilcoxon signed rank test. BMI, body mass index

## Discussion

Our study showed that more than half of the HCPs were overweight or obese. This finding is similar to that of other local studies among HCPs in Malaysia.^14,15^ A study conducted in the United States also reported that HCPs had a significantly higher risk of becoming obese than workers in other industry categories.^16^ The high percentage of obesity among HCPs is likely attributed to the long working hours, unhealthy diet, job stress and poor self-perception about body weight.^5,14,17,18^

In our study, the drivers and administrative clerks had the highest prevalence of obesity among all job categories, which is dissimilar to other local and international reports, where nurses contributed to the highest prevalence.^5,14,19^ Our findings could be explained by the sedentary nature of the work of drivers and clerks. Generally, our study is similar to a study conducted in India, which found the prevalence of obesity to be significantly lower among nurses than among doctors.^20^ Taken together, occupational categories may have an impact on obesity among HCPs.^5,14,19,20^ Accordingly, weight reduction programmes targeting more drivers and administrative clerks may be needed in the future.

Sex disparities in excess weight gain could be attributed to different eating patterns. Women tend to prefer dairy products or foods high in added sugars (e.g. cookies, chocolate and ice cream), while men tend to prefer meat-based products with greater protein content and that do not affect fat mass.^21,22^ This supports our results that the female HCPs had a higher prevalence of obesity or overweight than the male HCPs, also similar to most other reports.^17,23-25^ However, our results differ from those of other local studies that discovered men to have a higher prevalence of obesity.^14,15^

Our study intervention focusing on education and diet did not have a significant impact on body weight, which is similar to the findings of a previous meta-analysis.^26^ We found that 42.2% of the HCPs had improvements in their body weight after 6 months; however, it was not statistically significant. Despite this, we postulate that awareness of the importance of maintaining a healthy body weight had been instilled to nearly half of them, and hence, effort was made for weight loss. HCPs often deal with patients who are overweight and obese; thus, they play an important role in the prevention and management of obesity,^27^ as most patients will take action if provided with counselling regarding obesity management.^28^ Therefore, awareness of a healthy body image among HCPs is important in such situations.

Another plausible reason for the insignificant weight loss seen among our HCPs after the dietary approach and educational programme alone may be attributed to their unhealthy eating behaviours and the unsupportive eating environment in our health clinic during the movement control order (MCO) for the COVID-19 pandemic.^29^ Individuals with obesity tend to show a significantly higher preference for take-out food over home-cooked food.^30,31^ A supportive eating environment in a health clinic^32^ and dietary adherence^33^ are two key factors contributing to behavioural change and weight loss. In our programme, the nutritional advice given to the participants was more focused on the Malaysia Healthy Plate, involving advice to reduce sugary drinks and fatty food intake and increase high fibre intake. This involves a need to change daily eating habits, whether in the form of food portions or cooking methods. However, during the MCO, the mushrooming of food delivery services has enabled easy access to various types of unhealthy food. The increase in food delivery services and the lack of time to prepare home-cooked meals owing to added workload during the COVID-19 pandemic likely contributed to an unhealthy eating environment among the HCPs. This was noted during the monthly dietary counselling by the nutritionists. However, our study did not evaluate the knowledge, attitude and practice of the HCPs before and after the intervention.

According to a randomised controlled trial,^13^ a weight management programme combining diet, physical exercise and cognitive behavioural training was more successful in controlling weight than a monthly oral presentation. As such, a regular exercise schedule was initially planned as part of the FATS programme. However, physical distancing measures during the COVID-19 pandemic have deterred the continuation of the exercise programme. Most HCPs at Simpang Health Clinic also served as front liners (n=31), and this reduced their physical activities during working hours. The Malaysian Obesity Task Force also noted that at baseline, Malaysians have a sedentary lifestyle, and their energy costs (kcal/min) of habitual activities are lower than those of Caucasians.^32^ The implementation of the MCO further impeded outdoor physical activities among HCPs.^29^ This may be one of the reasons contributing to 58% of our HCPs gaining weight 6 months after the intervention.

In our study, younger age, pharmacist job and male sex were associated with higher success rates in weight loss. This finding can be useful in future weight reduction programmes where individuals with these factors can be utilised as small group leaders championing weight loss initiatives. Improvisations are proposed to increase the effectiveness of the FATS programme. The FATS programme consisted only of a series of health education sessions and dietary advice. It did not involve any counselling or physiotherapy owing to limited availability. This was deemed inadequate to induce behavioural change for sustained weight loss. A counsellor or psychologist can implement a cognitive behavioural approach to increase the success rate and consistency of weight loss^13,34^ Occupational therapists can provide guidance on occupational balance that can enhance incorporation of physical activities into daily routine, leading to sustainable behavioural changes.^35^ The involvement of family members may also enhance the efficacy of weight management programmes because family is the pillar of lifestyle modification and support.^36^ Thus, incorporating a broad-based multimodal approach in the FATS programme is likely to make it more successful.^13,33-36^

The limitation of this study is its small sample size. This was also part of the reason why we did not assess the participants’ readiness to lose weight, as it may further reduce the sample size of our study. Its design without a comparator arm limits the conclusions able to be drawn. Its conduct during the COVID pandemic also makes generalisation of the results to non-pandemic situations difficult.

## Conclusion

A weight loss programme solely consisting of a series of health discussion and nutritional advice is inadequate to induce weight reductions. A more broad-based multimodal approach, involving counsellors or psychologists and physiotherapists, may be considered in managing weight among HCPs.

**How does this paper make a difference in general practice?**

- Increase understanding of weight management program used in clinical practice.
- Suggests multimodal approach to be used in weight management program instead of health discussion and nutritional advice alone.
